# Prognostic Impact of Post-Diagnosis Smoking Cessation among Bladder Cancer Patients: A Systematic Literature Review and Meta-Analysis

**DOI:** 10.3390/cancers14164022

**Published:** 2022-08-20

**Authors:** Saverio Caini, Marco Del Riccio, Virginia Vettori, Giulio Francolini, Oriana D’Ecclesiis, Tommaso Cai, Aurora Gaeta, Guglielmo Bonaccorsi, Ines Zanna, Domenico Palli, Sara Gandini

**Affiliations:** 1 Cancer Risk Factors and Lifestyle Epidemiology Unit, Institute for Cancer Research, Prevention and Clinical Network (ISPRO), Via Cosimo il Vecchio 2, 50139 Florence, Italy; 2 Postgraduate School in Hygiene and Preventive Medicine, University of Florence, 50134 Florence, Italy; 3 Department of Health Sciences, University of Florence, 50134 Florence, Italy; 4 Radiation Oncology Unit, Azienda Ospedaliero-Universitaria Careggi, 50134 Florence, Italy; 5 Department of Experimental Oncology, European Institute of Oncology (IEO), IRCCS, 20141 Milan, Italy; 6 Department of Urology, Santa Chiara Regional Hospital, 38123 Trento, Italy

**Keywords:** bladder cancer, smoking cessation, survival, systematic review, meta-analysis

## Abstract

**Simple Summary:**

Cigarette smoking increases the risk of developing bladder cancer; many of these patients are active smokers at diagnosis. There is evidence that smokers with cancer at other sites (e.g., lung) may improve their chance of surviving if they stop smoking upon diagnosis. However, similar evidence is still lacking for bladder cancer. Here, we reviewed the scientific literature up to January 2022 but found a mere nine articles focusing on this topic, which provided insufficient evidence to draw firm conclusions. While more research is urgently needed, bladder cancer patients who are still smoking at diagnosis should receive information on the multiple health benefits of smoking cessation and all the support they need to stop successfully.

**Abstract:**

We reviewed the studies examining whether quitting smoking at or around diagnosis favourably affects the prognosis of bladder cancer (BC) patients, who are often active smokers at diagnosis. We found only nine eligible articles published until 31 January 2022, which encompassed around 5500 BC in total, the majority of which were nonmuscle invasive BC (only one paper included muscle-invasive BC). We used random effects meta-analysis to obtain a summary hazard ratio (SHR) and 95% confidence intervals (CI). The median proportion of smokers who quit at or around diagnosis was 29.8% (range 8.4–43.1%). For the overall, BC-specific, and progression-free survival, the studies were limited in number (*n* = 3) and provided conflicting results. At the same time, quitters did not appear to have a lower risk of recurrence than continued smokers (SHR 0.99, 95% CI 0.61–1.61). In conclusion, while the evidence is currently not sufficient to draw firm conclusions (especially for patients with muscle-invasive BC), physicians should not refrain from educating smoking BC patients about the benefits of smoking cessation and provide the necessary support.

## 1. Introduction

Bladder cancer (BC) is the twelfth most frequent cancer according to the GLOBOCAN estimates, with over 570,000 new cases diagnosed in 2020, and it causes 2.1% of all cancer-related deaths worldwide [1]. Cigarette smoking is the most important risk factor influencing BC risk [2], as it accounts for over one-third (36.8%) of all BC cases globally. However, with substantial variability by sex (the proportion is 43.7% and 15.2% among men and women, respectively) and geographically (with the highest attributable risk in Eastern and Central Asia, Eastern and Central Europe, and North Africa and the Middle East) [3]. BC risk increases by smoking duration and intensity, and there is evidence that quitting smoking reduces BC incidence, although the risk remains higher lifelong compared to never smokers [4,5].

Data from literature suggest that cigarette smoking also affects BC patient prognosis. Several clinical outcomes, including disease recurrence of nonmuscle invasive BC (NMIBC), progression to muscle-invasive BC (MIBC), and BC mortality, are more common among BC patients who smoke at diagnosis compared to never and former smokers [4,6,7]. A patient’s smoking history and current smoking status may not be actionable items for urologists, oncologists, and radiotherapists, who can only provide advice on smoking cessation during diagnostic work-out at the earliest, or once a BC diagnosis is made. Smoking cessation is challenging for cancer patients [8], and the need for smoking counseling may remain overlooked as clinicians give priority to surgery and systemic treatments. Thus, this may be a largely unmet clinical need, leaving plenty of room for improvement in the management of cancer patients [9]. Smoking cessation could represent a cost-effective and risk-free option to improve BC prognosis. Yet, there is surprisingly very limited evidence to date on whether smoking cessation after diagnosis benefits BC patients who are active smokers at diagnosis. We recently showed that stopping smoking at or around diagnosis is associated with substantial prognostic improvements (e.g., ≈20–30% longer overall survival (OS) compared to continued smokers) among patients with lung cancer [10], but comparable evidence is still lacking for BC. Aiming to fill this knowledge gap, and in the attempt to provide the background evidence for advocating the implementation of more structured approaches for smoking cessation in daily clinical practice, we performed a systematic review and meta-analysis on the association between smoking cessation at or around the diagnosis and the prognosis of BC patients.

## 2. Materials and Methods

This systematic review and meta-analysis were planned, conducted, and reported in compliance with the Preferred Reporting Items for Systematic Reviews and Meta-analysis (PRISMA) statement [11]. The review protocol was registered in PROSPERO (registration number CRD42021245560) prior to the start of our investigation [12].

### 2.1. Search Strategy

MEDLINE and EMBASE databases were searched for original articles published until 31 January 2022 that investigated the prognostic effect of quitting smoking at or around diagnosis (vs. continued smoking) among BC patients. In order to be as sensitive as possible and minimize the possibility of missing an eligible paper, we devised a search string that would encompass articles focusing on any cancer site, and only subsequently were the papers focusing on bladder cancer identified and checked for eligibility. Namely, the literature search was conducted by using the following string: (smok*) AND (cease OR cessation OR quit* OR stop*) AND (cancer OR carcinoma OR tumour OR malignancy) AND (survival OR prognos* OR outcome OR mortality). No time, geographical, or language restrictions were applied as long as an English abstract was provided.

### 2.2. Study Selection

Upon removing duplicates, the titles and abstracts of all retrieved papers were screened by three independent researchers (SC, MDR and VV). The articles not discarded at this initial stage were independently read in full copy by two author (VV and MDR) to decide on their eligibility; in case of disagreement, a consensus choice was taken with a third researcher (SC). Then, we checked eligible studies, previously published reviews, and meta-analyses for further relevant references. Studies were eligible if they provided (or allowed us to calculate) a hazard ratio (HR) and 95% confidence intervals(CI). They were also eligible if they provided measures of statistical uncertainty (like standard errors, variance, or exact *p*-values) to quantify the effect of quitting smoking at or around diagnosis. Such measures included the BC patient overall survival (OS), BC-specific survival, progression-free survival (PFS), or recurrence-free survival (RFS). In this review and meta-analysis, we defined “quitters” as BC patients who stopped smoking at diagnosis, early before it (up to 12 months before), or at some point afterward (e.g., before or during treatment). Studies in which the timing at smoking cessation for those labelled as quitters was not specified or did not match the above criteria were not eligible for inclusion in the present review. Publications not presenting original findings (e.g., commentaries, editorials, and letters without data) were also excluded.

### 2.3. Data Extraction

Two independent researchers (VV and MDR) extracted data from each eligible study and entered into an internally piloted spreadsheet the following information: country(-ies) and year(s) in which the study was conducted; study design; total number and demographic characteristics of smoking BC patients, along with their breakdown into quitters and continued smokers; exact definition of quitters and continued smokers; distribution of BC patients in terms of tumour stage and treatments received; follow-up length (median/mean and minimum/maximum); details on statistical analysis and variables used for adjustment if any. The HR and 95% CI for the association between at/around-diagnosis smoking status and patients’ survival were inverted if necessary in order to make the “continued smokers” the reference group (thus, HRs below 1.00 indicate a longer survival for quitters and vice versa for HRs above 1.00). Then, they were transformed into log(HR) and corresponding variance using Greenland’s formula [13]. The method by Parmar et al. [14] was applied in order to extract unadjusted HR and 95% CI from survival curves (if available in the text) when the authors directly provided no HR.

In terms of how results were presented, studies could be distinguished into two types: those in which quitters and continued smokers were compared directly, and those in which two separate HRs were presented that compared the survival of quitters and continued smokers to the third group of patients (e.g., never smokers), taken as the reference group. Studies falling into the latter category were considered eligible. Their characteristics and findings were tabulated and commented on in the text but could not be included in meta-analysis models (unless an unadjusted HR could be extracted from survival curves) nor used to quantify the degree of heterogeneity of HRs across studies (see below). This is because no measure of statistical uncertainty (e.g., standard errors) could be obtained for the direct comparison of quitters and continued smokers.

### 2.4. Quality Assessment

The Quality in Prognosis Studies (QUIPS) tool was used to assess the quality and bias in each included study [15].

### 2.5. Statistical Analysis

Meta-analysis was conducted if there were log (HR) for the direct comparison of quitters vs. continued smokers available from at least three independent papers, which was the case only for RFs (see Results). Summary hazard ratios (SHR) were calculated by fitting random effect models with maximum likelihood estimation, and 95% CI were obtained by assuming an underlying t distribution [16]. Heterogeneity was quantified using the I^2^ statistics, which can be considered the proportion of the overall variability across studies attributable to actual heterogeneity rather than chance [17]. Leave-one-out sensitivity analysis was performed to identify possible sources of the observed heterogeneity when I^2^ exceeded 50% [18]. Study characteristics that were planned a priori to be used in subgroup analysis and meta-regression included the study country and year of publication, patients’ age and sex, the exact timing of smoking cessation (strictly at or after diagnosis vs. up to 12 months before diagnosis), and whether the HR estimates were adjusted for potential confounders. The Egger’s and Begg’s tests were used to check for publication bias [19,20], and in case either test achieved statistical significance, we applied the “trim and fill” method to estimate the number of missing (i.e., unpublished) studies and provide an adjusted SHR [21]. For endpoints other than RFs (i.e., overall, bladder cancer-specific, and progression-free survival), the number of independent studies reporting HR for the direct comparison of quitters and continued smokers was lower than three (see Results). Thus, no formal meta-analysis and ancillary analyses (quantifying heterogeneity of the I^2^ statistics, searching for its causes, and detecting publication bias) could be conducted.

Statistical analyses were conducted using SAS software, version 9.4 (SAS Institute Inc., Cary, NC, USA) and R software, version 4.1.2. All tests were two-sided, and p-values were considered statistically significant when below 0.05.

## 3. Results

**Figure 1:**
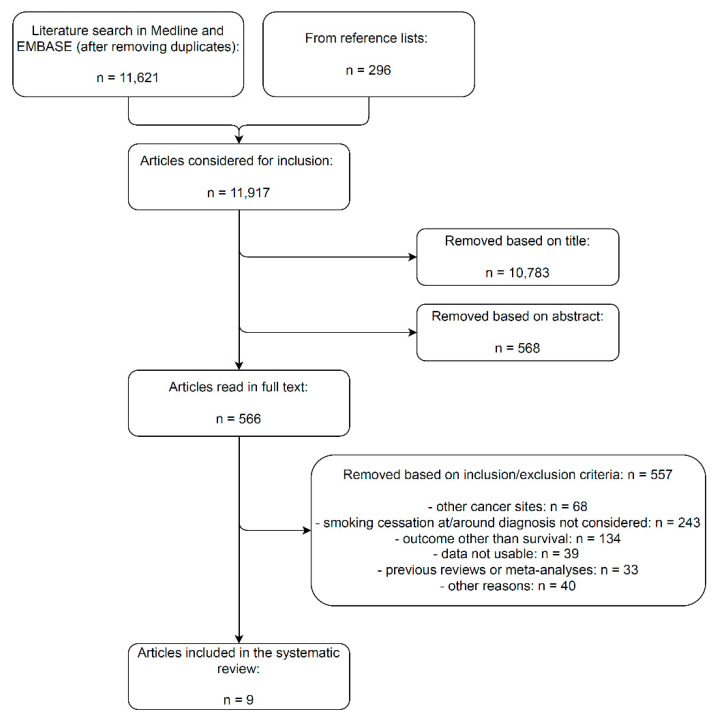
Flow-chart of the literature search and article selection for the systematic review and meta-analysis on the effect of quitting smoking at or around diagnosis on the survival of bladder cancer patients.

The literature search in Medline and EMBASE (until 31 January 2022) and the perusal of reference lists yielded a total of 11,917 non-duplicate entries (Figure 1). The number of articles that were discarded based on their title and abstract was 10,783 and 568, respectively, while 566 articles were read in full text and checked for eligibility. Of these, 557 did not match inclusion criteria, the most frequent causes for exclusion being the inability to consider smoking at/around diagnosis smoking cessation and the focus on outcomes other than survival endpoints. Of the articles that were excluded, two deserve an additional explanation. The study by Karlsson et al. was excluded because the post-diagnosis smoking status (quitter/continued) was neither self-reported by the patients nor ascertained through biological methods (e.g., exhaled CO or urinary/salivary cotinine) but rather inputted via deep learning algorithms [22]. Instead, the study by Furberg et al. was excluded because continued smokers were compared to ever smokers that were abstinent after BC diagnosis; the latter group is a mix of patients who quit post-diagnosis and patients who had stopped long before BC diagnosis [23].

Only nine articles were finally found eligible and included in the systematic review [24,25,26,27,28,29,30,31,32]: these were published between 1999 and 2021 and encompassed a total of 5490 BC patients (ranging across studies from a minimum of 107 to a maximum of 1733) (Table 1). All studies’ mean/median age at BC diagnosis fell in the seventh or eighth decade of life. Men made up just over two-thirds of the totality of all patients. Among BC patients that were smokers at diagnosis, the proportion of those who quit ranged between 8.4% and 43.1%. Except for Lee et al. [29], all studies included only patients with NMIBC (information on tumor stage was not available in Koshiaris et al. [26] and Tao et al. [28]). Studies also differed in treatments administered to patients, particularly regarding the proportion of those treated with intravesical BCG immunotherapy, radiotherapy, and chemotherapy. Patients’ median/mean follow-up varied across studies between 3.2 and 6.7 years. In most studies, the quitters’ category included only smokers who had stopped smoking at diagnosis or up to 1 year after that, while in three studies, patients who had quit smoking up to 1 year prior to diagnosis were included among quitters (Table 2). In the study by Tao et al., a time-dependent definition of post-diagnosis smoking status was defined and used for the analyses. We entered the results of this analysis in the Tables below (instead of the analysis comparing those who had never smoked vs. had never stopped smoking after diagnosis). Of note, the determination of at/around diagnosis smoking status relied on self-reporting in all studies. At the same time, exhaled carbon monoxide (CO) or cotinine concentration in urine or saliva was never used to verify cessation or monitor continuous abstinence. The latter represented the most common limitation of the included studies (Supplementary File S1): other potential sources of bias were the lack of details on the % of patients lost to follow-up (and whether they differed from those who remained in the study), and some lack of systematicity concerning the reporting of the study results.

The association between post-diagnosis smoking cessation and overall and BC-specific survival was examined in only three studies for each of the two health outcomes (Table 3). Compared to continued smokers, smoking cessation was found to be beneficial in terms of OS in the studies by Tao et al. (HR 0.34, 95% CI 0.13–0.92) and by Sfakianos et al. (reference category: never smokers; HR 1.03, 95% CI 0.63–1.68 for continued smokers, and 0.64, 95% CI 0.31–1.34, for quitters), while no association emerged in Koshiaris et al. Concerning BC-specific survival, results were mixed, with a prognostic advantage for quitters being reported in the study by Sfakianos et al., while Lee et al. detected no difference, and Koshiaris et al. reported a non-significant 25% increase in the risk of dying from BC among quitters compared to continued smokers (Table 3). No meta-analysis was performed because of the small number of studies.

**Figure 2:**
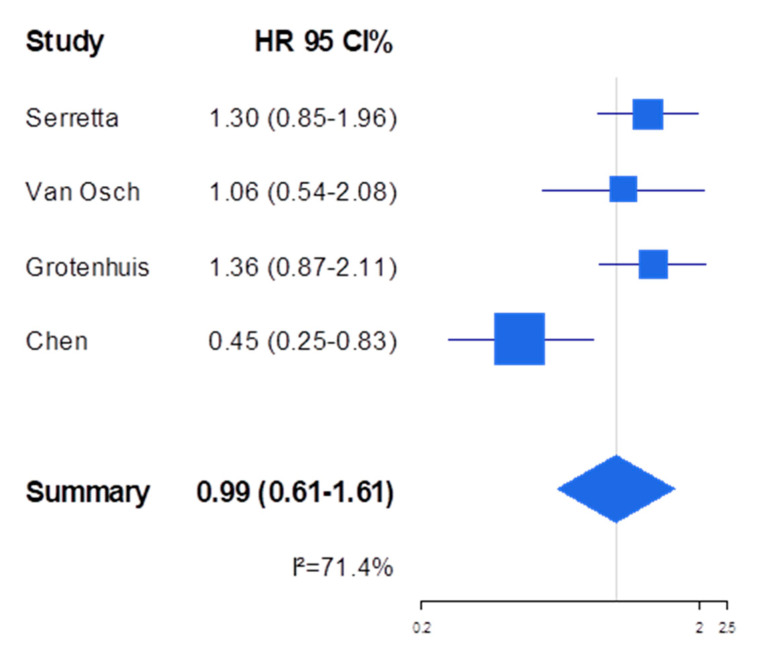
Forest plot for the association between quitting smoking at or around diagnosis (vs. continued smoking, taken as reference) and the risk of recurrence among bladder cancer patients. HR: hazard ratio. CI: confidence intervals.

The association between smoking cessation at or around diagnosis and PFS among BC patients was examined in only three studies, which did not consistently demonstrate a prognostic advantage for either quitters or continued smokers (Table 4). Instead, seven independent studies reported on the association between post-diagnosis smoking cessation and RFS (Table 4). Of these, the four most recent studies [24,25,27,29] found an increased risk of recurrence among quitters. In comparison, in the three oldest studies [30,31,32], the association was in the opposite direction (achieving statistical significance in Chen et al.). Meta-analysis was conducted by entering in the model the four papers that provided or allowed us to calculate an HR for the comparison of quitters and continued smokers [24,25,27,31]: the association with BC patients’ RFS was not significant (summary HR 0.99, 95% CI 0.61–1.61) and flawed by high heterogeneity (I^2^ = 71%, which dropped to 0%, however, when removing the study by Chen et al.) (Figure 2), but with no evidence of publication bias (*p*-value: Begg’s test = 0.75, Egger’s test = 0.27). The summary estimates obtained upon excluding the study by Chen et al. confirmed the lack of effect of smoking cessation on RFS (1.28, 95% CI 0.97–1.68). Because of the limited number of studies that could be included in the meta-analysis, it was not possible to conduct subgroup analysis or meta-regression to formally explore the potential source of heterogeneity across studies.

## 4. Discussion

To our knowledge, this is the first attempt to systematically review and meta-analyse the studies focusing on the impact of smoking cessation at or around diagnosis on the prognosis of BC patients. We found only nine papers matching our inclusion criteria, which encompassed almost 5500 patients, the vast majority of which were NMIBC (only one article included patients with MIBC). The limited number of available studies, and the limitations affecting some of them, made it impossible to answer our study question and warn against drawing firm conclusions on any effect of smoking cessation on the survival of BC patients (this applies especially to patients with MIBC). In general, we did not find very suggestive evidence that smokers who decide to quit during the diagnostic work-up or upon BC diagnosis have a better prognosis than those who continue to smoke thereafter. However, we believe it appropriate to carefully weigh the strength of the evidence available for the different endpoints that we considered in our review, as well as what emerged from the assessment of study quality and susceptibility to bias.

Regarding the impact of smoking cessation on local relapse, the meta-analysis showed a lack of significant benefit for quitters compared to continued smokers. The studies that could not be entered in the meta-analysis were consistent with those findings. Similar to what emerged for local relapse, there was no convincing indication that smoking cessation upon diagnosis or shortly before it may substantially affect the progression-free, overall, or disease-specific survival of BC patients. However, it must be recognized that the quantity and quality of the evidence for each of all these four endpoints were rather poor and definitely insufficient to draw firm conclusions, mainly due to the very limited number of articles that could be included in the review (only three for all but recurrence-free survival, which even prevented meta-analysis). This was further exacerbated by the unavailability of data for directly comparing continued smokers and quitters in several included studies. This made them ineligible for inclusion in formal meta-analysis (including quantification of heterogeneity). Moreover, it should be noted that the power to detect the effect of smoking cessation on OS or disease-specific survival may have been curbed by the inclusion in most studies of a majority of NMIBC, a clinical entity indeed associated with frequent relapses but with a generally moderate direct impact on patient’s survival [33]. In particular, studies that mostly include patients with a generally good prognosis would require to be appropriately sized and with a suitably long follow-up length in order to be able to detect any prognostic impact of smoking cessation (or any other intervention) on survival, and this may not have been the case for all of the studies that were included in our review. Conversely, MIBC is a more aggressive disease characterized by a substantial risk of distant metastases and a worse prognosis [33]. The impact of post-diagnosis smoking cessation (or other prognostic factors) on survival might be better studied in this scenario. Moreover, it cannot be ruled out that the impact of quitting may differ between smokers with NMIBC vs. MIBC. Unlike NMIBC, routine treatment options for MIBC include radical cystectomy, concurrent radio-chemotherapy, or chemotherapy containing platinum doublets [34,35,36], i.e., treatments with a considerable burden on the patient (much higher than that of treatments used for nonmuscle invasive disease), so that continued smoking, which may aggravate underlying smoke-related comorbidities (like chronic obstructive pulmonary disease, cardiovascular diseases, or renal failure), may limit tolerability forcing to recur to de-escalated therapy. Moreover, considering the significant impact that hypoxia modification may have on response to radiotherapy for BC treatment [37], whether a distinct influence of smoking cessation on clinical outcomes varies across different treatment options would be an additional study question to investigate in future research efforts. Thus, additional well-designed prognostic studies encompassing patients with both NMIBC and MIBC, with a suitably long follow-up, a more reliable method to ascertain one’s changes in smoking habits (e.g., via repeated exhaled CO or salivary cotinine measurements), and detailed information on tumour and patients’ characteristics (including how these were treated), are warranted in order to attempt to give a reliable answer to the scientific question we had set ourselves.

In this systematic review and meta-analysis, the overall lack of convincing evidence and, therefore, the inability to answer its research question depended both on the limited number of available studies (which, in addition, prevented a formal investigation of possible sources of heterogeneity of estimates across studies using subgroup analysis and meta-regression). Moreover, some key intrinsic limitations in terms of study quality and availability of results, the most important of which is the fact that, except for RFS, the other endpoints of interest (OS, BC-specific survival, and PFS) were neglected in the majority of studies. As already mentioned, most studies explored outcomes in populations of (all or mostly) NMIBC patients, which may be suboptimal considering their generally good prognosis and, therefore, the need of long observation periods to detect a significant prognostic benefit of smoking cessation. Moreover, there was a paucity of information on recurrence and quality of life among patients with NMIBC, which only rarely (≈10%) progress to MIBC, while this may be important clinically considering that smoking cessation may lead to the reduced need for adjuvant therapy and subsequent cystoscopic examinations. There was broad heterogeneity in the definition of the prognostic factor under study (smoking cessation), and its ascertainment relied entirely on self-reporting, which may have further hampered the possibility of detecting a favourable effect on BC patients’ survival. Finally, post-diagnosis smoking status was not defined (except in Tao et al.) as a time-varying exposure, which has further potential for misclassification and biasing the results.

## 5. Conclusions

In conclusion, the available evidence was insufficient to draw firm conclusions on whether smokers diagnosed with BC may reduce their risk of disease relapse or progression or extend their survival by stopping smoking upon diagnosis. Despite the current lack of evidence on this specific topic, smoking cessation remains a cost-effective, risk-free strategy that undoubtedly has a far-reaching positive effect on treatment feasibility (especially when the more aggressive disease is considered), risk of subsequent second primary tumours, other smoking-related illnesses (and prognosis thereof), and overall quality of life [38,39]. Hence, smokers who did not cease upon BC diagnosis should be referred by their treating physicians (e.g., urologists, oncologists, and radiation oncologists, as well as their general practitioners) to smoking cessation services and counselling, given the several benefits that this can bring to their health and quality of life.

## Figures and Tables

**Table 1:** Main characteristics of the studies included in the systematic review and meta-analysis on the prognostic effect of quitting smoking at or around diagnosis on the survival of bladder cancer patients.

Author, Year	Country	Sex (% Men)	Age (Years)	No. Patients ^(a)^	Smoking Status			Years of Diagnosis	Tumour Stage	Treatments	Follow-Up (Years)
					Non-Smokers at Diagnosis ^(b)^	Continued Smokers	Quitters				
Serretta, 2021 [24]	Italy	NA	Median 63 (range 57–70)	194	0 (0.0%)	127 (65%)	67 (35%)	2008–2012	Ta (57%), T1 (37%), Tis (6%)	TUR (100%)	Median 5.7 (range 0.5–4.4)
van Osch, 2018 [25]	UK	79%	Median 71 (IQR 63–77)	722	519 (72%)	186 (26%)	17 (2%)	2005–2011	pTa (66%), pT1 (33%), pCis (1%)	TUR (100%)	Median 4.2 (IQR 2.7–5.0)
Koshiaris, 2017 [26]	UK	74%	Mean 66.9	1733 ^(c)^	0 (0.0%)	850 (49%)	356 (21%)	1999–2013	NA	surgery (9%), CT (15%), RT (4%)	Na
Grotenhuis, 2015 [27]	Netherlands	82%	Mean 63.3 (range 25–92)	963 ^(d)^	671 (70%)	194 (20%)	71 (7%)	1995–2010	Ta (69%), T1 (26%), Cis (4%), unknown (1%)	TUR only (46%), adjuvant CT (32%), adjuvant IT (21%), adjuvant CT + IT (1%), other or unknown (<0.01%)	Median 3.7 (IQR 2.7–4.7)
Tao, 2013 [28]	China	100%	Na	107 ^(e)^	58 (54%)	NA	NA	1986–2010	NA	NA	Mean 5.3
Lee, 2012 [29]	South Korea	90%	Mean 62.2	597	337 (56%)	159 (27%)	101 (17%)	1989–2008	≤T2 (57%), >T2 (43%)	radical cystectomy (100%)	Median 4.7 (range 0.2–18.9)
Sfakianos, 2010 [30] ^(f)^	USA	68%	Median 76	623	485 (77%)	97 (16%)	41 (7%)	1994–2008	Ta (35%), T1 (35%), Tis (30%)	TUR + intravesical BCG (100%)	Median 6.7
Chen, 2007 [31]	Taiwan	100%	Median 67 (range 36–90)	265	128 (48%)	78 (30%)	59 (22%)	1997–2005	Ta (62%), T1 (38%)	TUR (100%), chemotherapy (58%), BCG (19%)	Median 3.2 (range 0.3–9.4)
Fleshner, 1999 [32] ^(f)^	USA	80%	Mean 61.2 (range 29–85)	286	127 (44%)	108 (38%)	51 (18%)	1985–1995	Ta (52%), T1 (31%), Tis (17%)	TUR (100%), initial BCG therapy (23%)	Mean 4.8 (range 0.2–10.9)

^(a)^ This refers to the patients included in the analyses aimed at estimating the effect of at/around smoking cessation on cancer survival or recurrence (it may be lower than the total number of patients in the study). ^(b)^ This category includes never smokers and long former smokers. ^(c)^ For 527 patients (30% of the total), the smoking status during the first year of follow-up (continued/quitter) was unknown and imputed via multiple imputations. ^(d)^ The smoking status (continued/quitter) of 27 patients who were smoking at diagnosis was not known. ^(e)^ The breakdown of the 49 active smokers at diagnosis (46%) into continued smokers and quitters was not specified. ^(f)^ The studies by Sfakianos et al. 2010 and Fleshner et al. 1999 were conducted at the same hospital (Memorial Sloan-Kettering Cancer Center, New York, USA) and included bladder cancer patients’ diagnoses during 1994–2008 and, respectively, from 1985–1995, so a minor overlapping of the study samples cannot be ruled out. NA: not available. TUR: transurethral resection. CT: chemotherapy. RT: radiotherapy. IT: immune therapy. BCG: bacillus Calmette-Guérin.

**Table 2:** Definition of quitters and continued smokers in the studies included in the systematic review and meta-analysis on the prognostic effect of quitting smoking at or around diagnosis on the survival of bladder cancer patients.

Author, Year	Quitters	Continued Smokers
Serretta, 2021 [24]	Definitively stopped smoking at diagnosis.	Continued to smoke after diagnosis or restarted smoking after a period of cessation.
van Osch, 2018 [25]	Quit smoking post-diagnosis and abstained consistently.	Continued smoking post-diagnosis.
Koshiaris, 2017 [26] ^(a)^	Had stopped smoking the last time, the smoking status was assessed during the first year of follow-up.	Continued smoking the last time the smoking status was assessed during the first year of follow-up.
Grotenhuis, 2015 [27]	Quit smoking in the first year after diagnosis.	Did not quit smoking within 1 year after diagnosis
Tao, 2013 [28]	Never smoked cigarettes after diagnosis.	Continued to smoke until death or the latest follow-up interview.
Lee, 2012 [29]	Quit smoking between 1 year and 1 month prior to diagnosis.	Smoked between 1 year and 1 month prior to diagnosis.
Sfakianos, 2010 [30]	Stopped smoking at the time of the start of treatment.	Continued smoking after diagnosis.
Chen, 2007 [31]	Stopped smoking within a year before and 3 months after diagnosis.	Never stopped smoking even at 3 months after diagnosis.
Fleshner, 1999 [32]	Quit smoking between 1 year prior to and up to 3 months following the diagnosis.	Continued smoking after diagnosis.

^(a)^ For 527 patients (30% of the total), the smoking status during the first year of follow-up (continued/quitter) was unknown and was imputed via multiple imputations.

**Table 3:** Hazard ratio (HR), 95% confidence intervals (CI), and details of the statistical analysis for the association between at/around diagnosis smoking status (cessation/continuation) and bladder cancer overall and bladder cancer-specific survival.

Author, Year	Patients Group (According to Smoking Status)	HR	95% CI	Variables Used for Statistical Adjustment	Exclusion of Events within Predetermined Time from the Start of Follow-Up
**Overall survival**					
Koshiaris, 2017 [26]	continued smokers	1.00 (ref.)		Age, sex, treatment, other	Yes (deaths within the first annual follow-up after diagnosis)
	Quitters	1.02	0.81–1.30		
Tao, 2013 [28] ^(a),(b)^	Smoking cessation (time-varying)	0.34	0.13–0.92	Age, cumulative smoking, treatment, other	Yes (deaths within the first annual follow-up after diagnosis)
Sfakianos, 2010 [30]	Never smokers	1.00 (ref.)		Age, sex, tumour stage	No or not mentioned
	Continued smokers	1.03	0.63–1.68		
	Quitters	0.64	0.31–1.34		
**Bladder cancer-specific survival**					
Koshiaris, 2017 [26]	Continued smokers	1.00 (ref.)		Age, sex, treatment, other	Yes (deaths within the first annual follow-up after diagnosis)
	Quitters	1.25	0.71–2.20		
Lee, 2012 [29]	Never smokers	1.00 (ref.)		Age, tumour stage and grade, other	No or not mentioned
	Continued smokers	1.17	0.64–2.13		
	Quitters	1.17	0.78–1.75		
Sfakianos, 2010 [30]	Never smokers	1.00 (ref.)		Age, sex, tumour stage	No or not mentioned
	Continued smokers	1.27	0.64–2.52		
	Quitters	0.80	0.30–2.18		

^(a)^ The HR and corresponding 95% CI were inverted to make continued smokers the reference category. ^(b)^ The HR comparing those who had never smoked after diagnosis (quitters) and those who had never stopped after diagnosis (continued smokers) was 0.06 (95% CI 0.01–0.44). HR: hazard ratio. CI: confidence intervals.

**Table 4:** Hazard ratio (HR), 95% confidence intervals (CI), and details of the statistical analysis for the association between at/around diagnosis smoking status (cessation/continuation) and bladder cancer recurrence-free and progression-free survival.

Author, Year	Patients Group (According to Smoking Status)	HR	95% CI	Variables Used for Statistical Adjustment	Exclusion of Events within Predetermined Time from the Start of Follow-Up
**Progression-free survival**					
Grotenhuis, 2015 [27]	Continued smokers	1.00 (ref.)		Age, cumulative smoking, tumour stage and grade, treatment, other	No or not mentioned
	Quitters	1.24	0.55–2.80		
Sfakianos, 2010 [30]	Never smokers	1.00 (ref.)		Age, sex, tumour stage	No or not mentioned
	Continued smokers	1.16	0.65–2.08		
	Quitters	0.81	0.35–1.88		
Chen, 2007 [31] ^(a)^	Continued smokers	1.00 (ref.)		None (unadjusted HR extracted from Kaplan-Meier curve)	Yes (progression within 8 weeks of diagnosis)
	Quitters	0.40	0.14–1.16		
**Recurrence-free survival**					
Serretta, 2021 [24] ^(a)^	Continued smokers	1.00 (ref.)		Age, cumulative smoking, tumour stage and grade, other	No or not mentioned
	Quitters	1.30	0.85–1.96		
van Osch, 2018 [25] ^(b)^	Never smokers	1.00 (ref.)		Age, sex, tumour stage and grade, other	No or not mentioned
	Continued smokers	1.04	0.65–1.66		
	Quitters	1.47	0.63–3.41		
Grotenhuis, 2015 [27]	Continued smokers	1.00 (ref.)		Age, cumulative smoking, tumour stage and grade, treatment, other	No or not mentioned
	Quitters	1.36	0.87–2.11		
Lee, 2012 [29]	Never smokers	1.00 (ref.)		Age, tumour stage and grade, other	No or not mentioned
	Continued smokers	0.85	0.59–1.21		
	Quitters	0.96	0.64–1.44		
Sfakianos, 2010 [30]	Never smokers	1.00 (ref.)		Age, sex, tumour stage	No or not mentioned
	Continued smokers	1.04	0.77–1.40		
	Quitters	0.75	0.49–1.16		
Chen, 2007 [31] ^(a)^	Continued smokers	1.00 (ref.)		Cumulative smoking, tumour stage, treatment, other	Yes (any recurrence within 8 weeks of diagnosis)
	Quitters	0.45	0.25–0.83		
Fleshner, 1999 [32]	Former smokers	1.00 (ref.)		Age, sex, tumour stage and grade, treatment, other	Yes (any recurrence within 3 months of surgery)
	Continued smokers	1.40	1.03–1.91		
	Quitters	0.99	0.77–1.25		

^(a)^ The HR and corresponding 95% CI were inverted to make continued smokers the reference category. ^(b)^ The HR and 95% CI shown in the table are for the time-to-first recurrence. The HR and 95% CI for the risk of multiple recurrence events is 1.10 (0.72–1.69) for continued smokers and 0.85 (0.35–2.04) for quitters (compared to never smokers, taken as reference). HR: hazard ratio. CI: confidence intervals.

## Supplementary Materials

The following supporting information can be downloaded at: https://www.mdpi.com/article/10.3390/cancers14164022/s1, Supplementary File S1 (spreadsheet): Quality assessment of the included studies using the QUIPS (Quality in Prognostic Studies) tool.
